# Pigmentary keratitis in pugs in the United Kingdom: prevalence and associated features

**DOI:** 10.1186/s12917-019-2127-y

**Published:** 2019-10-30

**Authors:** S. Maini, R. Everson, C. Dawson, Y. M. Chang, C. Hartley, R. F. Sanchez

**Affiliations:** 1 Langford Veterinary Services, University of Bristol, Langford, Bristol, UK; 2 North Downs Specialist Referrals, Bletchingley, UK; 3 The Royal Veterinary College, Ophthalmology Service, Department of Clinical Science and Services, University of London, North Mymms, Herts, UK; 4 Research Office, The Royal Veterinary College, Camden Campus, London, UK; 5 Ophthalmology Service, Specialistische Dierenkliniek Utrecht (part of Anicura), Utrecht, The Netherlands

**Keywords:** Entropion, Brachycephalic, Canine, Cornea, Pigment, Keratitis, Corneal pigmentation, Medial entropion

## Abstract

**Background:**

Pigmentary keratitis (PK) is commonly recognised in Pugs, but its aetiology is not completely understood. The aim of this study was to determine the prevalence and associated features of PK in Pugs in the United Kingdom (UK).

**Results:**

A total of 210 Pugs (420 eyes) were recruited from 12 UK dog shows and social events. The median age of Pugs recruited was 2.50 years (range 0.25–16.25 years). Pigmentary keratitis was detected in 369/420 (87.8%) eyes and in at least one eye 193/210 (91.9%) Pugs, of which 17/193 (8.8%) were affected unilaterally and 176/193 (91.2%) bilaterally. Pigmentary keratitis was typically mild to moderate (46.3 and 49.9% of eyes, respectively). Detection of PK was significantly associated with increased age (*P* = 0.002) and the presence of medial entropion of the lower eyelid (MELE) (*P* = 0.001). Severity of PK was significantly associated with the grade of MELE (*P* < 0.001). There was also a correlation between the presence of limbal pigment and PK (*P* = 0.036) that warrants further study.

**Conclusions:**

This study estimated a high disease prevalence of PK in UK Pugs, and demonstrated significant associations with age and the presence of MELE. These associations, which have not been previously reported, offer an insight into the underlying pathophysiology of this condition in Pugs. The results encourage further population research, such as prospective longitudinal studies. These findings also support the development of clinical and breeding strategies based on the reduction of MELE and, possibly, limbal pigment.

## Background

Pigmentary keratitis is a term used to describe the development of corneal pigmentation associated with chronic inflammation [1]. If PK encroaches upon the visual axis, it can cause significant visual impairment and, in severe cases, blindness [2, 3]. Pigmentary keratitis occurs due to centripetal migration of melanocytes from the limbal and perilimbal region and subsequent deposition of melanocytic pigment within the corneal epithelium and anterior stroma [1, 3–6]. Corneal pigmentation is also frequently reported as a feature of inflammatory corneal pathology, such as keratoconjunctivitis sicca (KCS), chronic superficial keratitis (pannus) and chronic, ulcerative/nonulcerative keratitis [2, 7–12]. Pigmentary keratitis appears to develop more rapidly and readily in some brachycephalic breeds and it has been shown to be widespread within the Pug breed in two studies based in the United States of America (USA) and one study from Austria, that reported estimated prevalence rates of 82.4, 71.8 and 70%, respectively [1, 11, 13]. Reputed causative or contributory factors of PK in Pugs include chronic irritation from distichiasis, nasal fold trichiasis, medial entropion, and macroblepharon [14, 15]; however, supporting evidence for their influence on the development of PK has so far proven elusive [11, 13]. Additional suggestions have been made of possible primary components in the development of PK in the Pug breed, such as a limbal stem cell deficiency or genetic factors [13, 16, 17]. Pugs are a popular breed in the UK, with the number of Pugs registered with the UK Kennel Club (KC) having doubled between 2009 and 2015; figures plateaued at approximately 10,000 Pugs per year between 2014 and 2018.

The aim of this study was to contribute to the body of research on this poorly understood but widespread condition by estimating the prevalence of PK in Pugs in the UK, and determining if there were any statistical associations with ocular, adnexal or facial features.

## Results

### Study population

**Fig. 1:**
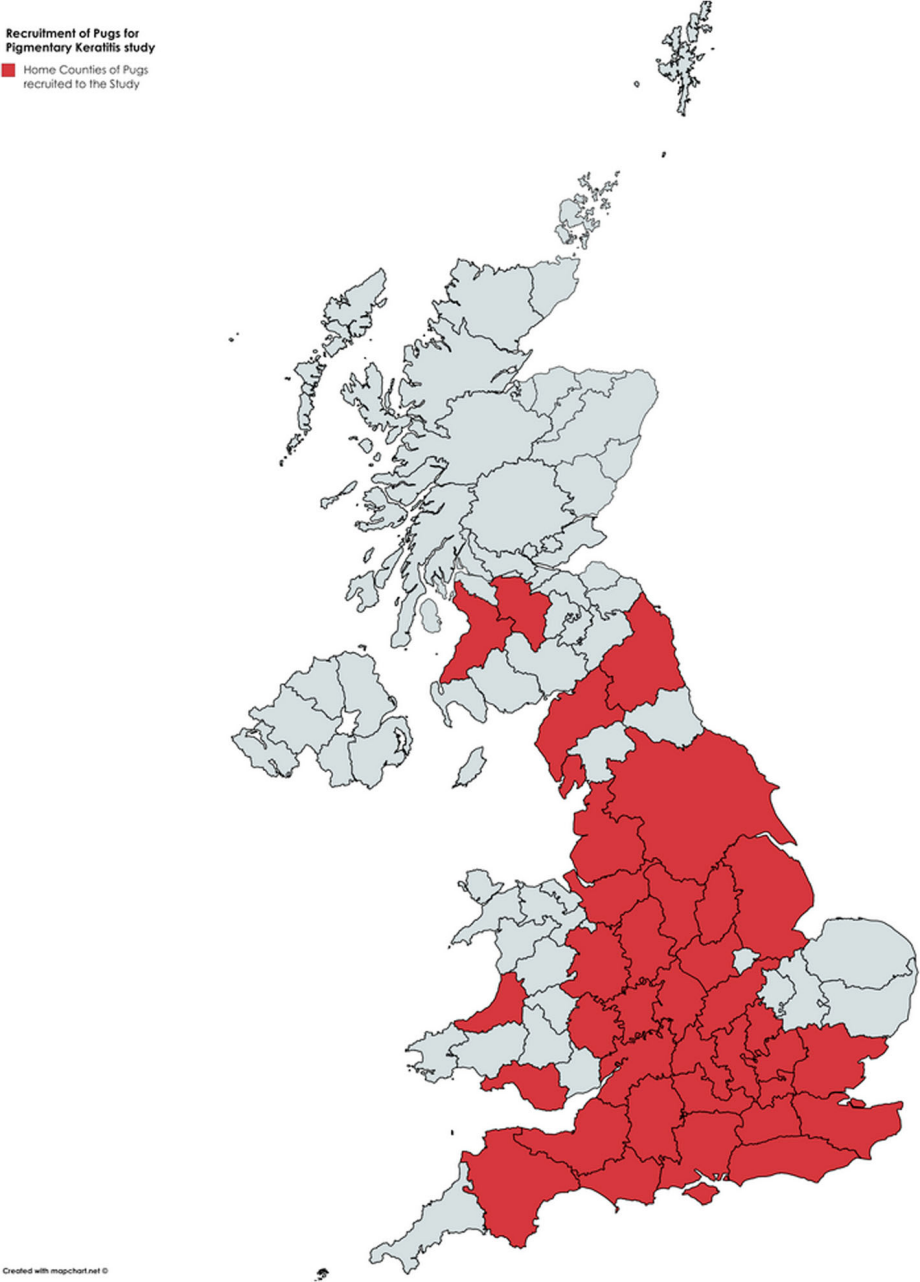
Recruitment of Pugs within the United Kingdom. Map created using MapChart https://mapchart.net (licensed under a Creative Commons Attribution-Share Alike 4.0 International License)

Two hundred and ten dogs, with a total of 420 eyes, were included in the study. Individuals were recruited from one of 12 national events and, collectively, represented a large area of the UK (Fig. 1). Sex, neuter status, age, coat colour, UK KC registration and show status were as follows for those dogs where this information was provided/recorded. Sex was known for 208/210 (99.0%) dogs; the sample population included 120/208 (57.7%) females and 88/208 (42.3%) males. Neuter status was provided for 206/210 (98.1%) dogs; a total of 66/206 (32.0%) dogs were neutered and 140/206 (68.0%) were entire. Age was known for 203/210 (96.7%) Pugs; the median age was 2.50 years (interquartile range 2.92 years, range 0.25–16.25 years). Coat colour was recorded for 207/210 (98.6%) cases. Colour variations were categorised into three groups: fawn (146/207; 70.5%), black (50/207; 24.2%) and other (11/207; 5.3%). Most dogs for whom registration status was provided (173/210; 82.4%) were registered with the UK KC (158/173; 91.3%); 15/173 (8.7%) were not registered. Show status was provided for 131/210 (62.4%) dogs; a total of 81/131 (61.8%) were described as ‘show dogs’ and 50/131 (38.2%) were not routinely entered into shows.

### General ocular, adnexal and facial features (excluding PK)

Features that were not associated with PK detection or severity included medial entropion of the upper eyelid (MEUE), craniofacial index (CFI), over-nose-wrinkle (ONW), nasal fold width, palpebral fissure length, iris-to-iris persistent pupillary membranes (IIPPMs) and distichia (Table 1). MELE, which was associated with PK severity, could be assessed in 374/420 (89.0%) eyes. A total of 352/374 (94.1%) eyes exhibited MELE and 22/374 (5.9%) did not. Of the 374 eyes that were assessed, 276/374 (73.8%) were categorised as grade 1 MELE and 76/374 (20.3%) as grade 2; and comprised 78.4 and 21.6% of the eyes with MELE, respectively. Length (i.e. %) of lower eyelid affected by MELE ranged from 0.0–50.0%, with a mean (+/−*sd*) of 22.4 (+/− 9.9) %. IIPPMs were detected in 198/420 (47.1%) eyes and were not detected in 222/420 (52.9%) eyes; they were detected in at least one eye of 117/210 (55.7%) dogs, leaving 93/210 (44.3%) that were unaffected. Distichiasis was detected in 30/420 (7.1%) eyes and was not detected in 390/420 (92.9%) eyes. At least one eye of 25/210 (11.9%) dogs was affected by distichiasis, with most dogs being unaffected (185/210; 88.1%). A total of 170/210 (81.0%) dogs were examined to see if they had an ONW; an ONW was present in 59/170 (34.7%) dogs and was absent in 111/170 (65.3%) dogs. Medial entropion of the upper eyelid was present in 28/410 (6.7%) eyes. Craniofacial index was assessed in 206/210 (98.1%) dogs; values ranged from 0.05 to 0.33, with a mean of 0.18 (+/− 0.04). Nasal fold width was assessed in 399/410 (95.0%) eyes; values ranged from 1.0 to 24.0 mm, with a mean of 5.83 (+/− 2.06) mm. Palpebral fissure length was measured in 363/410 (86.4%) eyes; values ranged from 18.0 to 30.0 mm, with a mean of 23.96 (+/− 2.17) mm. All Pugs in this study (420/420 eyes) had a Schirmer tear test 1 (STT1) test performed to rule out with certainty that there was no KCS; measurements ranged from 15.0 to 33.0 mm/min of wetting, with a mean of 21.10 (+/− 3.24) mm/min. A total of 102/420 eyes had STT1 readings for less than 1 min (15–35 s); 94/102 had STT1 readings of at least 30 s. All 102 eyes were included in the study as they measured at or above 15 mm of wetting.

**Table 1:** Univariable analysis of detection and severity of PK and association with predictors

Predictor	Detection of PK	Severity of PK
	OR (95% CI), *P*	OR (95% CI), *P*
Age (per year)	1.53 (1.16–2.00), 0.002	1.08 (0.98–1.20), 0.132
Sex (male vs. female)	1.44 (0.66–3.15), 0.362	0.96 (0.59–1.56), 0.878
Neuter status (neutered vs. entire)	0.70 (0.31–1.52), 0.355	1.02 (0.60–1.73), 0.947
KC registration (registered vs. no)	1.20 (0.32–4.53), 0.792	0.84 (0.31–2.32), 0.738
Show dog status (yes vs. no)	0.50 (0.18–1.40), 0.188	0.63 (0.34–1.2), 0.162
STT1 measurement	1.00 (0.93–1.07), 0.919	0.98 (0.92–1.05), 0.573
Limbal pigmentation	0.95 (0.87–1.04), 0.277	1.04 (0.98–1.11), 0.183
MELE (grade)		
Grade 1 vs. MELE not present	6.31 (2.18–18.26), 0.001	0.62 (0.21–1.81), 0.380
Grade 2 vs. MELE not present	13.97 (3.44–56.81), < 0.001	1.94 (0.59–6.41), 0.279
Grade MELE not present vs. 2	0.07 (0.02–0.29), < 0.001	0.52 (0.16–1.71), 0.279
Grade 1 vs. Grade 2	0.45 (0.17–1.20), 0.112	0.32 (0.18–0.58), < 0.001
MELE (% length)	1.02 (0.98–1.06), 0.279	1.02 (0.99–1.04), 0.135
MEUE (% length)	1.01 (0.97–1.05), 0.694	1.02 (0.99–1.04), 0.168
CFI (< 0.18 vs ≥0.18)	1.45 (0.-3.27), 0.370	0.91 (0.55–1.49), 0.707
ONW (yes vs no)	1.75 (0.60–5.07), 0.306	1.27 (0.74–2.16), 0.386
Nasal fold width (per mm)	1.27 (1.03–1.56), 0.023	1.07 (0.88–1.30), 0.482
Palpebral fissure length (per mm)	1.08 (0.92–1.27), 0.343	1.08 (0.95–1.21), 0.235
IIPPMs (presence vs absence)	0.69 (0.39–1.22), 0.206	0.96 (0.63–1.46), 0.853
Distichia (presence vs absence)	0.49 (0.23–1.06), 0.069	1.22 (0.61–2.44), 0.580

### Pigmentary keratitis – descriptive statistics

Pigmentary keratitis was detected in at least one eye of 193/210 (91.9%) dogs. Seventeen out of 193 (8.8%) had unilateral PK and 176/193 (91.2%) exhibited bilateral PK. A total of 369/420 (87.9%) eyes were affected by PK. Of those affected eyes, 171/369 (46.3%) were classified as mild, 184/369 (49.9%) as moderate, and 14/369 (3.8%) as severe.

### Pigmentary keratitis – detection

Detection of PK was not significantly associated with signalment, breed club registration, ophthalmic, or any of the adnexal and facial predictors other than age and MELE (Table 1) in the univariable analysis. There was insufficient data to assess coat colour as a predictor for PK detection. Both age (OR = 1.76, 95%CI 1.31–2.36, *P* < 0.001) and presence of MELE (grade 1 vs 0: OR = 9.98, 95%CI 3.12–31.94, *P* < 0.0001; grade 2 vs 0: OR = 13.19, 95% CI 3.30–52.76, *P* < 0.001) remained significant in the multivariable analysis. No significant interaction between MELE and age on PK detection was observed (*P* = 0.09).

### Pigmentary keratitis – severity

Due to the relatively low number of severe cases, the moderate and severe groups were amalgamated to create a combined moderate/severe group, for the purposes of repeated measures logistic regression analysis (mild vs. moderate/severe). Severity of PK was not associated with signalment, breed club registration, or any of the ophthalmic, adnexal or facial predictors other than MELE (Table 1). Increasing PK severity was significantly associated with higher grade MELE (OR = 0.32, 95% CI: 0.18–0.58, *P* < 0.001, when comparing grade 1 to grade 2). No significant interaction between MELE and limbal pigmentation on PK severity was observed (*P* = 0.666).

As an association of PK with limbal pigment was not found (*P* = 0.183), the authors included an additional group of 16 Pugs from outside the study population to test if a larger population would help increase statistical power of this calculation, enough to find statistical significance; the results showed that the inclusion of the additional cases offered enough statistical power to demonstrate a statistically significant association between the severity of PK and increased limbal pigmentation (OR = 1.07, 95% CI: 1.00–1.15, *P* = 0.036) in the multivariable analysis.

## Discussion

This study suggests there is a high prevalence of PK in Pugs living within the UK. The authors selected a representative sample of the national population by attending a wide variety of breed shows and social events. The prevalence of PK within the sample population was higher than reported in similar studies performed in the USA and Austria [1, 11, 13]. This may reflect differences in population, breeding patterns, genetic background, previous veterinary attention or other as yet unknown factors.

The significant association of PK with the presence of MELE and its severity is, to the authors’ knowledge, the first report confirming an association between PK in Pugs and this conformational abnormality, which has long been suspected to be a contributory factor in this disease. This is in contrast with a previous study, which did not demonstrate a statistically significant influence of MELE on PK in Pugs [13]. This disparity may be attributed to differences in population and/or in study design, or to type I or II statistical errors. It is interesting to view the results of the present study in the context of a recent publication which demonstrated microscopic inflammatory changes within corneas affected by PK [1]. The significant associations between PK and MELE found in the current study could be explained by the irritative effect of MELE on the medial cornea of Pugs. This study also found that PK was significantly associated with increased age. The association with age was not entirely surprising as, clinically, PK is known to progress over time [16]. It seems logical to conclude that if corneas with PK have inflammatory changes [1] and if severe entropion is associated with PK (which worsens with age), that surgical correction of severe MELE would have a positive effect on the long-term corneal health of affected Pugs. It would also seem logical to consider the development of breeding strategies that focus on reducing the presence of severe MELE in Pugs. A large-scale prospective cohort study tracking the progression of PK in the presence of MELE, and in Pugs that have undergone surgical MELE correction would undoubtedly further clarify the impact of the associated features identified in the present study. Early, prototype studies have hinted that the correction of MELE, possibly with additional medical therapy, is an area of interest for further research [9, 18, 19]. However, until the results of future investigative studies are available, owners of Pugs with PK and severe MELE should be informed of the associations found to date, as they may wish to consider options that decrease ocular surface inflammation, such as surgical MELE correction.

When the authors realized that an association of PK with limbal pigment was not present, they included a group of 16 dogs from outside the study population solely to test if a larger population would help increase statistical power of this calculation. In doing so the authors found there was indeed a statistically significant association between the severity of PK and increased limbal pigmentation, which was not entirely surprising. This additional group could not be included in the larger analysis because they did not have an STT1 measured and therefore KCS could not be ruled out as the cause of PK. Pigmentary keratitis in Pugs is associated with the presence of MELE, as shown in the present study, but it can also be associated with KCS [11]. It seems logical to suspect an association between PK and the presence of limbal pigment in Pugs in the absence of KCS. However, the statistical strength of this association warrants further study if we are to determine how important the presence of limbal pigmentation is as a risk factor for the development of PK in Pugs. This study demonstrates that the population size needs to be large and offers the approximate number of Pugs needed with a normal STT1 reading and a clinical history free of KCS.

Previous population studies of Pugs reported on the presence of distichiasis as well as IIPPMs and CFI [11, 13, 20]. The present study corroborates the results of those papers in which no significant association between distichiasis and PK in Pugs was found [11, 13]. With regards to IIPPMs, one study reported a prevalence of 83.8% in the left eye and 85.3% in the right eye [13], while another study reported a much lower prevalence of 8.46% [11]. The prevalence of IIPPMs in this study was between the two previously published results. A variance in prevalence rates may be due to population differences, as each of the three studies was performed in a different country. The mean CFI of the Pugs included in the current study was higher than in a previous paper, which reported a CFI of 0.08 (+/− 0.01) [20]. Again, this could reflect differences between populations although it is possible that the presence of a large nasal fold hindered the accurate measurement of muzzle length in some cases, which may account in part for the difference between the two studies.

A limbal stem cell deficiency has been proposed as a possible cause of PK in Pugs [13, 17]. A confocal microscopy study of PK-affected Pug corneas supported the role of inflammation as opposed to a limbal stem cell deficiency [1]. However, the authors of that paper agreed that further studies were required to definitively rule out a possible role of stem cell deficiency in Pugs with PK [1]. Microscopic evaluation of affected Pug corneas to prove or disprove this theory was well beyond the scope of the present study. Genetic analysis has also been suggested as a potentially rewarding area of research [13]. However, due to the low number of unaffected Pugs (i.e. controls) identified in the present study, genetic analysis would appear to be challenging to pursue in the UK population.

Limitations of this study include failure to reach the ideal sample size as proposed by power analysis. Additionally, there was a low number of unaffected dogs. To compound this, all unaffected individuals were less than 5 years of age, likely because a large proportion of Pugs were recruited from shows. This has two important implications: a) the importance of this condition in the Pug population may be underestimated and b) it is unknown if young, unaffected dogs will remain free of PK throughout their lives. It is possible that the inclusion of Pugs that had previously had ulcerative keratitis might have had an impact on the estimated prevalence, as corneal pigmentation is known to develop secondary to chronic inflammation [2, 4, 7–12, 21], and Pugs have been shown to exhibit a high prevalence of corneal ulceration [22]. However, PK in Pugs starts classically in the medial cornea and has a centripetal progression in the shape of a triangle or wedge [3, 13] and, in the authors’ experience, corneal ulceration has a tendency to lead to a less predictable pigmentation pattern, as amorphous pigmented or non-pigmented scars tend to develop in the spot where a cornea was ulcerated. Yet, distinguishing between the two might not be possible and this is a potential problem of every PK study of Pugs. Even the exclusion of dogs with previously known ulcerative keratitis might not necessarily remove all cases that have had ulcerative keratitis because a small ulcer might go unnoticed by the owner, heal and scar. The examiners of this study made every effort to distinguish between the two presentations although it is acknowledged that the PK data collected might have been affected by pigmentation caused by previous ulceration.

## Conclusions

The prevalence of PK in UK Pugs in this study population was very high. Pigmentary keratitis was more likely to be detected in older Pugs and in those with limbal pigmentation and MELE, especially if MELE was severe. The results of this study offer an insight into the underlying pathophysiology of PK in Pugs and encourage further population research, such as prospective longitudinal studies, to further inform these findings. Moreover, these results support the development of clinical and breeding strategies on the reduction of MELE and limbal pigment.

## Methods

### Study design

A cross-sectional study design was selected, with the following objectives: to estimate prevalence and report descriptive statistics, and to investigate the presence or absence of statistical associations with suspected risk factors.

### Study population and methods

**Fig. 2:**
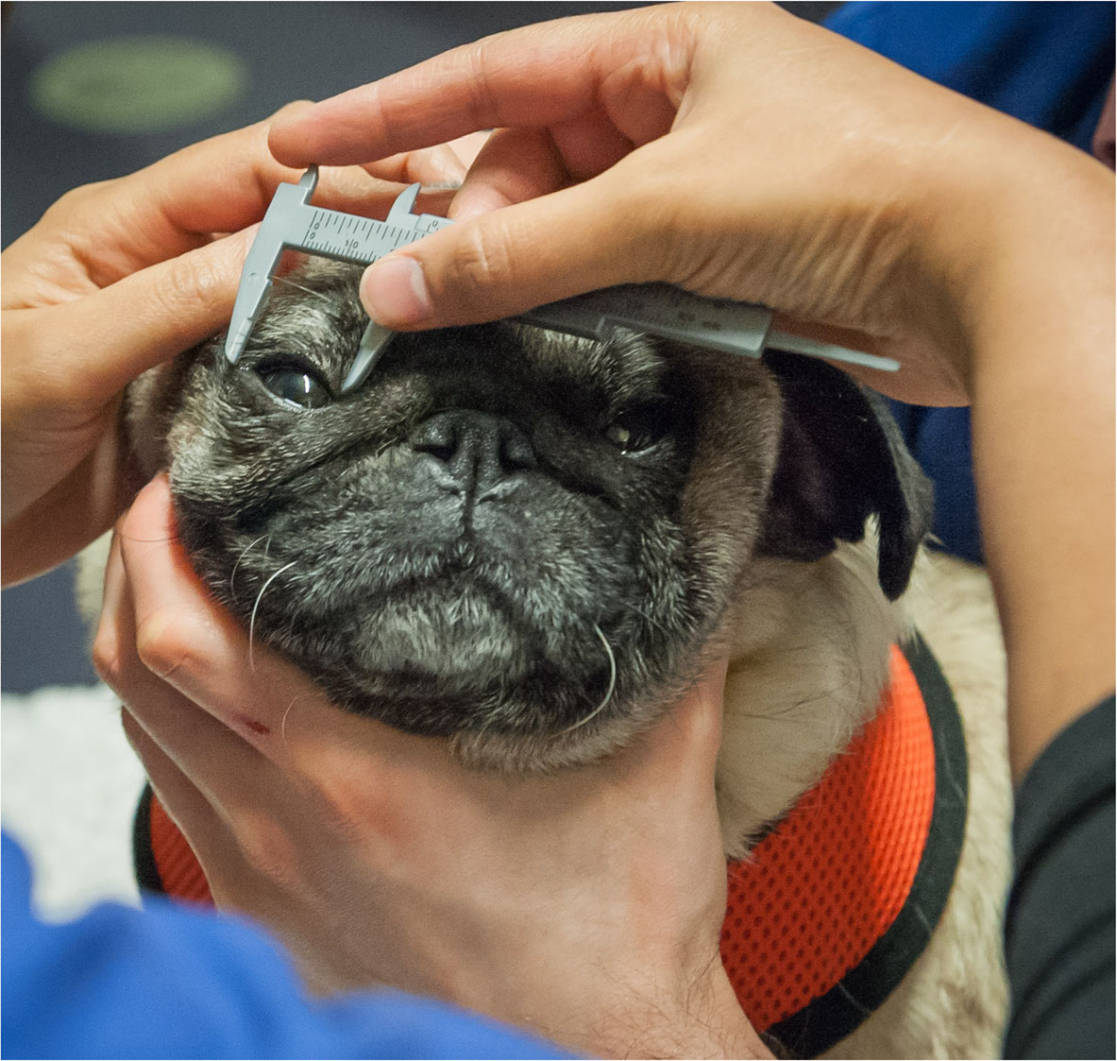
Measurement of unstretched palpebral fissure width with a blunt-ended Jameson caliper

All study methods were approved by the Royal Veterinary College (UK) Ethics & Welfare Committee. Informed, written consent was obtained from all participating owners. Pugs were enrolled and examined at one of 12 events (three breed club dog shows and nine social events e.g. ‘Pug parties’, dog charity ‘garden parties’, breeder/owner social gatherings) in nine different counties across the UK (East Sussex, Northamptonshire, Lincolnshire, Cheshire, Hertfordshire, London, Wiltshire, Gloucestershire and South Yorkshire) between July 2014 and October 2017. All Pugs that were presented to the examiner at these events were enrolled to the study, unless they had already been examined at a prior event. Pugs with a history of KCS were excluded from the study due to the documented link between KCS and corneal pigmentation [8, 11]. Signalment and ophthalmic history were obtained from owners by use of owner-completed questionnaires prior to examination. Examinations were performed free of charge and comprised slit-lamp biomicroscopy of ocular adnexa and the anterior segment, STT1, and ocular and facial morphometrics. An examination form (Additional file 1) was used to record examination findings. Ocular and facial measurements were collected using previously defined measuring protocols [23]. Craniofacial index was then calculated and used as a measure of brachycephaly [20]. The presence or absence of an ONW, MELE and MEUE were recorded. Additionally, unstretched palpebral fissure width (i.e. distance in millimetres between medial and lateral canthus), was measured using a blunt-ended Jameson caliper (Fig. 2), as was the nasal fold width (i.e. width in millimetres of nasal fold skin that could be grasped by the caliper). Ophthalmic examination was performed by one of two board-certified veterinary ophthalmologists (RFS & CH), primarily for assessment of PK. The presence of distichiasis and IIPPMs was also recorded. Ocular and facial morphometrics were collected by the same observer (SM).

### Schirmer tear test 1

The authors specifically inquired about an ophthalmic history of KCS and attempted to measure STT1 readings in every Pug; Pugs that did not tolerate STT1 measurement were excluded from the study. Inclusion in the study required that the Pug exhibited a moist ocular surface on ophthalmic examination. Obvious clinical signs of KCS (except for the presence of corneal pigmentation) were grounds for immediate exclusion, including one or more than one of the following: the presence of a dull ocular surface in the non-pigmented part of the cornea, presence of ulcerative keratitis, presence of mucous discharge that adhered to the ocular surface and/or moderate, marked or severe conjunctival hyperaemia.

### Assessment of pigmentary keratitis

**Fig. 3:**
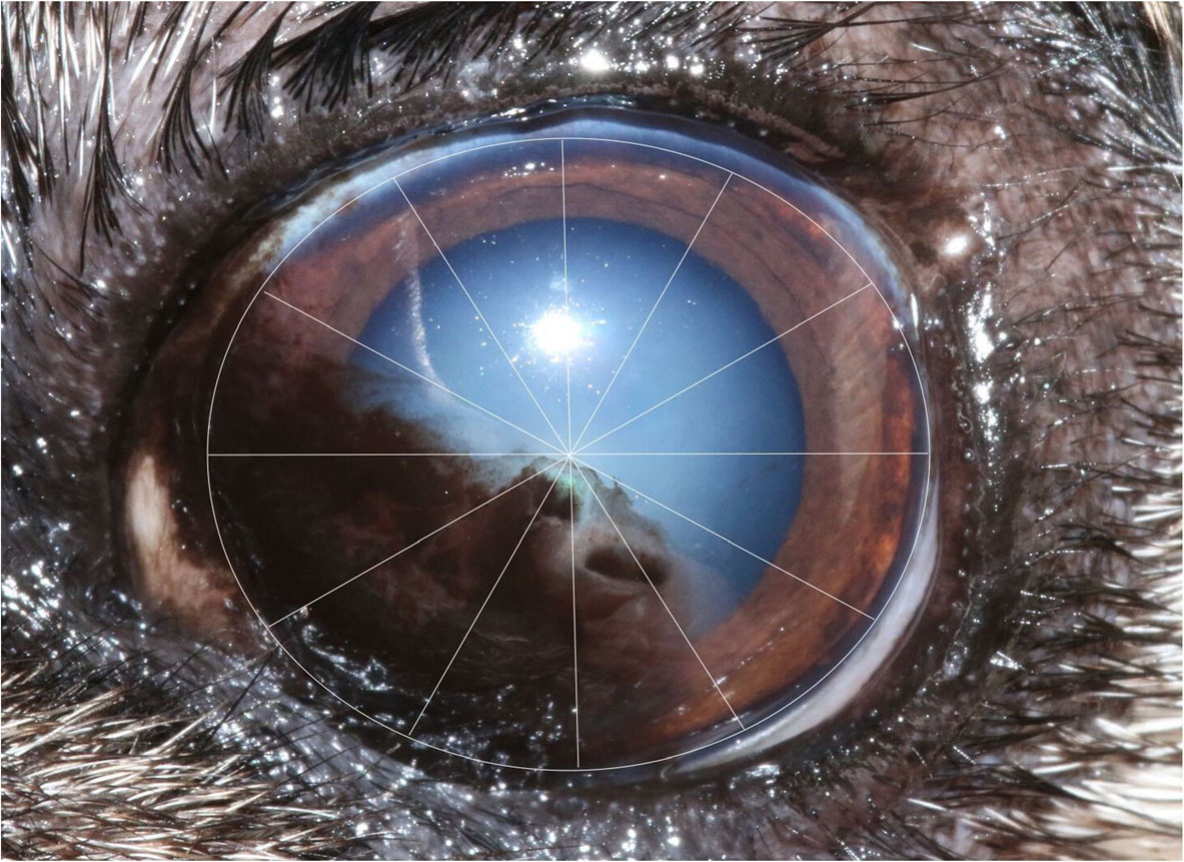
Assessment of corneal ‘clock hours’. Six clock hours were affected by corneal pigmentation (6 points). Two additional points were allocated as the pigment extended beyond the resting pupil edge. Total = 8 points

**Fig. 4:**
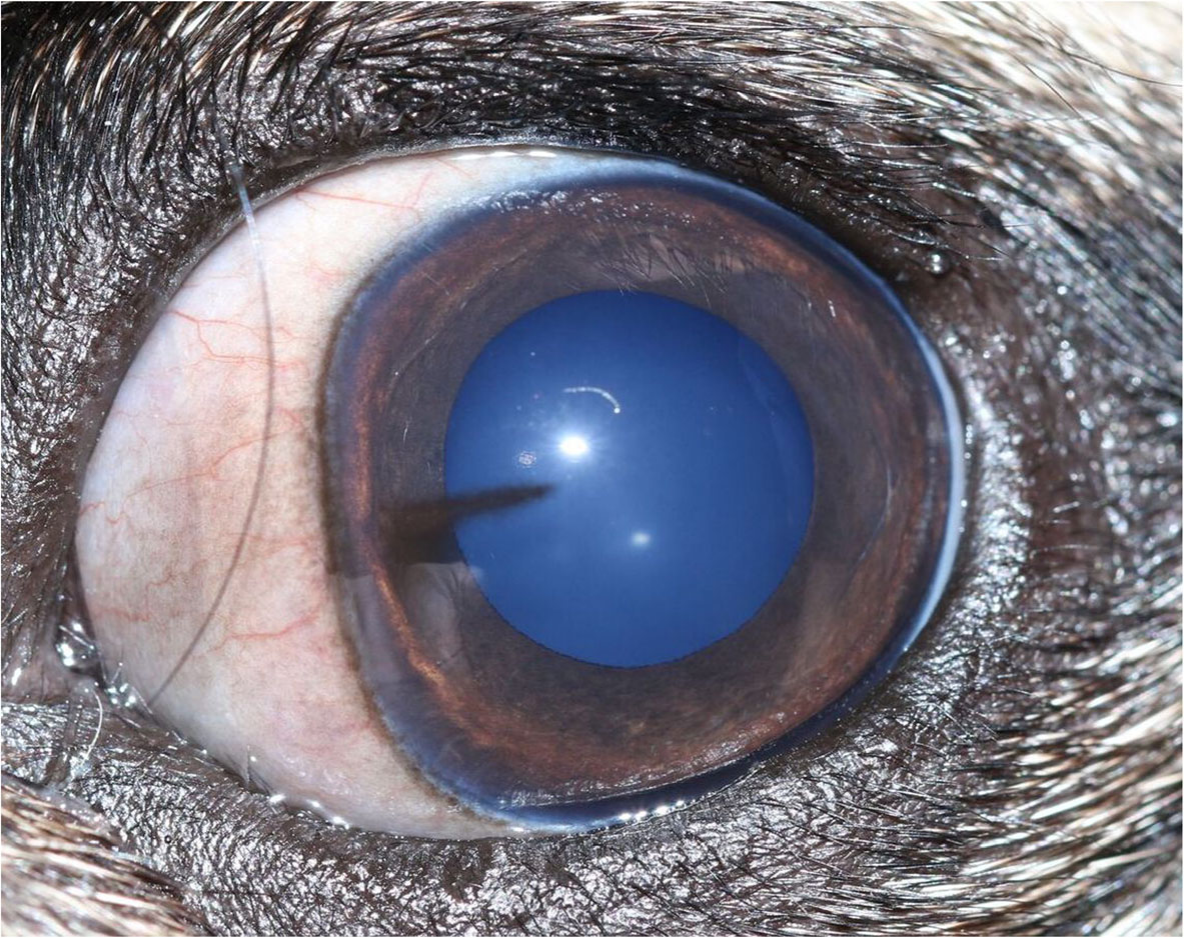
Assessment of pigment lines. Single lines of pigmentation were allocated half a point; two additional points were allotted as the pigmentation extended beyond the resting pupil edge. Total = 2.5 points

**Fig. 5:**
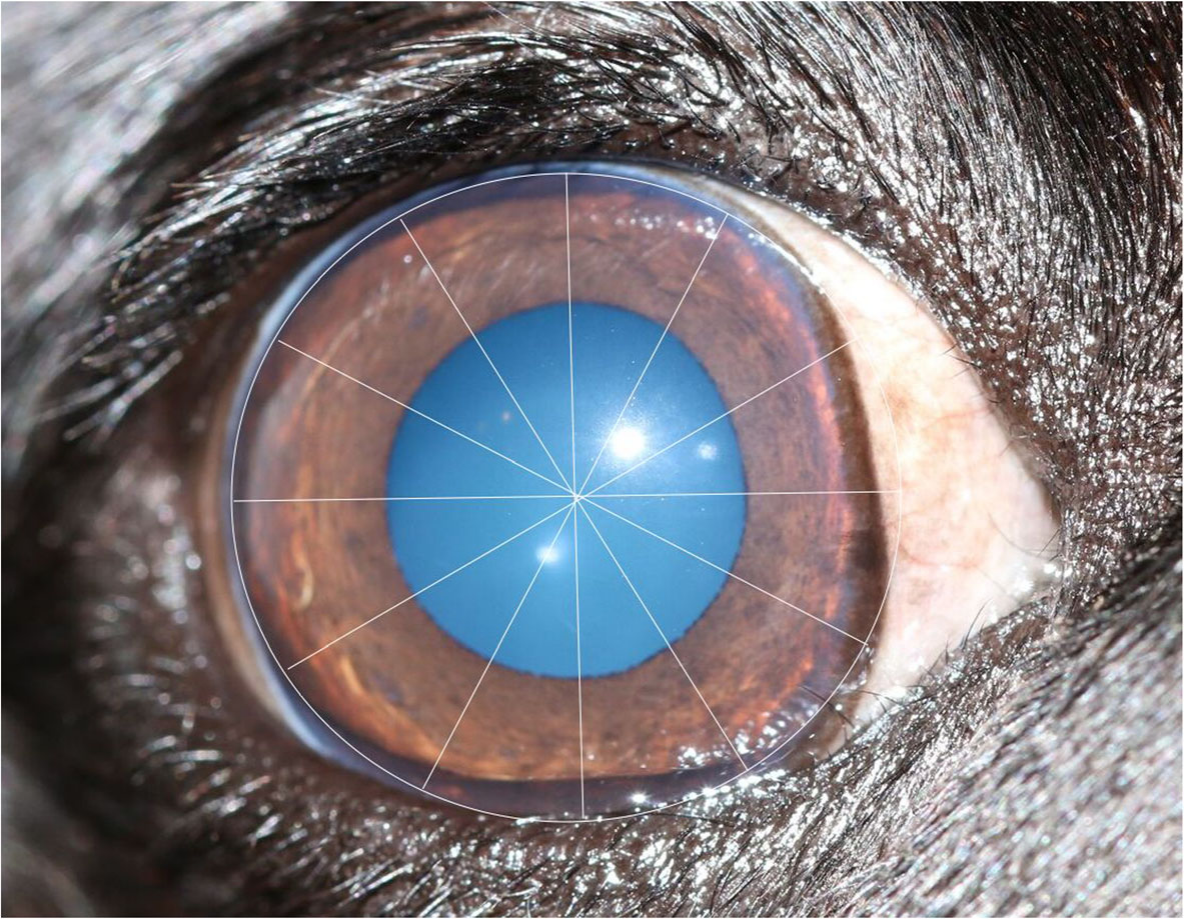
Assessment of limbal brush border. Corneal pigmentation extends just beyond the limbus creating a ‘limbal brush border’ affecting three clock hours (3 points). Total = 3 points

**Fig. 6:**
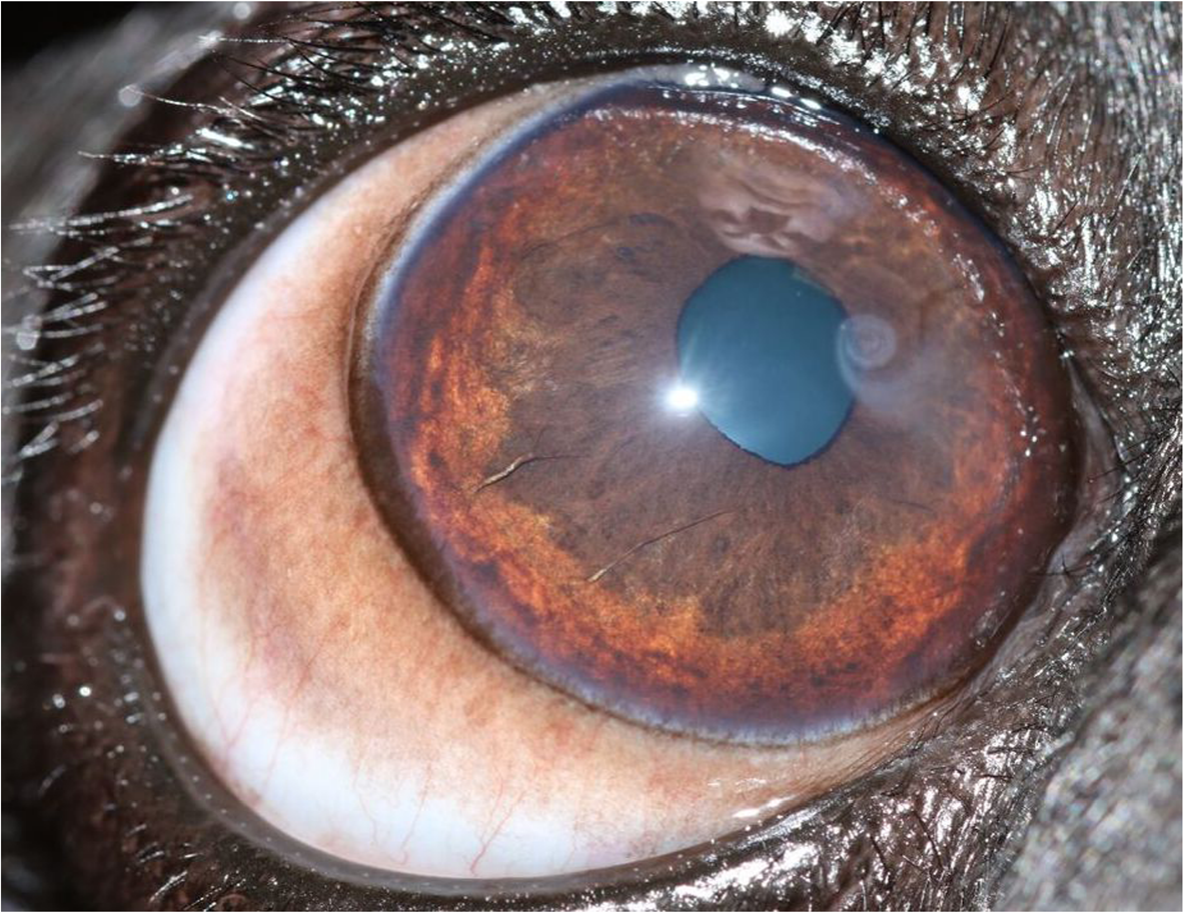
Assessment of grey/white corneal lesions and swirls. The swirl was allotted points as though it represented corneal pigmentation; one point per clock hour plus an additional two points for encroaching upon the visual axis. Laterally, a limbal brush border affects two corneal clock hours (2 points). Total = 5 points

**Fig. 7:**
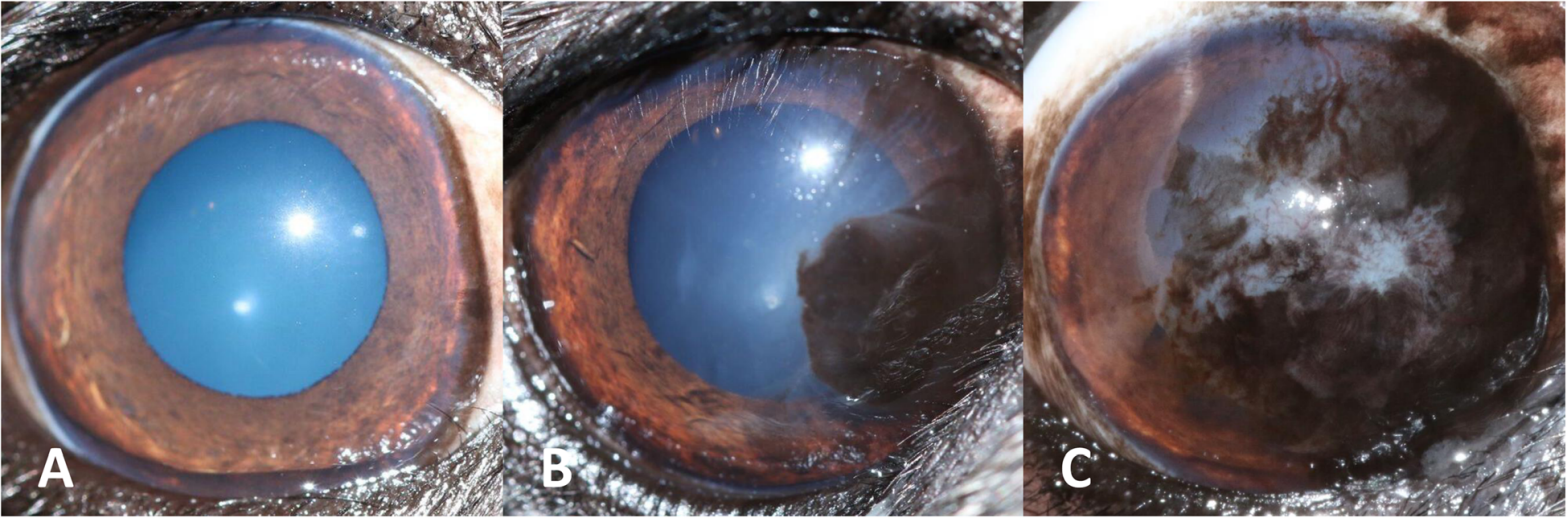
Severity of PK. Examples of mild (**a**), moderate (**b**) and severe (**c**) PK

A grading system for PK was developed by one of the authors (RS) for this study and was used for the assessment of all participants’ eyes. The corneal surface was divided into 12 sectors or ‘clock hours’. The extent of corneal pigmentation was assessed according to the number of ‘clock hours’ that were affected; one point was awarded per clock hour (Fig. 3). Single ‘lines’ of pigmentation were allocated a half-point (Fig. 4). One additional point was given if the pigmentation extended to the resting pupil edge; two additional points were assigned if the pigmentation extended beyond the resting pupil edge, encroaching upon the visual axis (Figs. 3 and 4). In cases where the corneal pigmentation extended only just beyond the limbus, this was termed a ‘limbal brush border’ and one point was allocated per clock hour affected (Fig. 5). Grey-white corneal lesions that presented medially and/or axially, often times with a centripetal ‘swirl’ appearance [3, 6], were considered a precursor to corneal pigmentation. These grey-white lesions were allotted points as though they represented corneal pigmentation i.e. one point was awarded per clock hour, with an additional 1–2 points if the swirl extended to or beyond the resting pupil edge (Fig. 6). The overall point score was then calculated and eyes were assigned to one of three groups; mild (0.5–4.5 points), moderate (5.0–9.5 points) and severe (10.0–14.0 points) PK (Fig. 7).

### Assessment of MELE

**Fig. 8:**
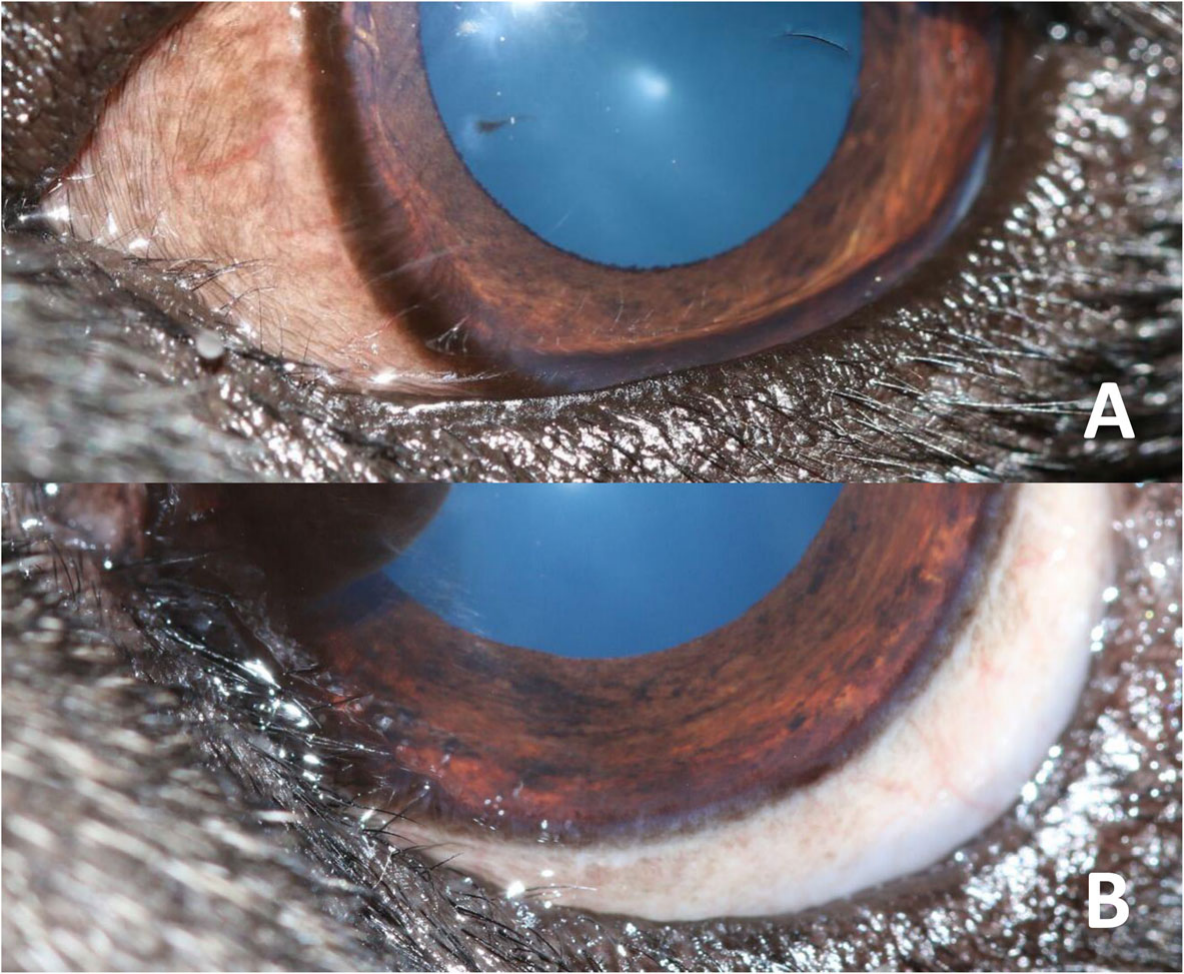
Assessing length of MELE. Examples of 25% lid length affected (**a**) and 33% lid length affected (**b**)

**Fig. 9:**
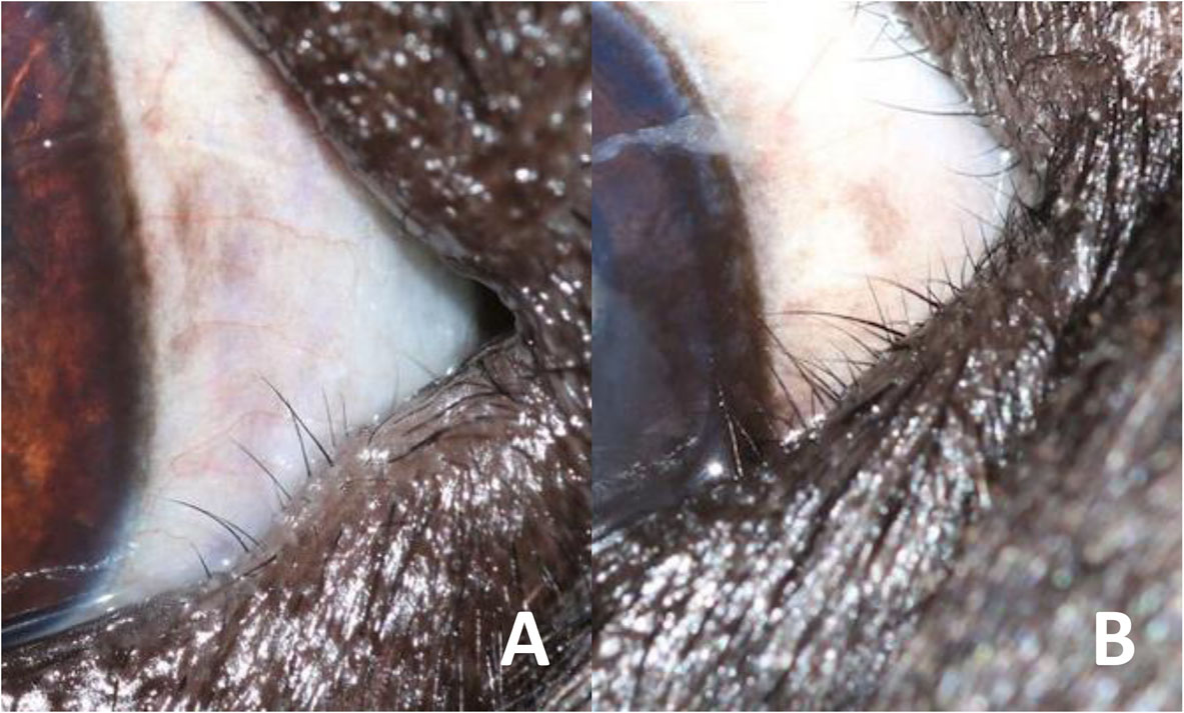
Assessing grade of MELE. Examples of grade 1 (**a**) and grade 2 (**b**) MELE

Three methods were used to record this data:

i) Presence or absence of MELE, which was recorded as a binary measure.
ii) Length of MELE. This was the proportion of eyelid length affected by MELE expressed as a percentage of lid length e.g. 25, 50% (Fig. 8). Medial entropion of the upper eyelid was also recorded in this manner.
iii) Grade of MELE. This was the severity of MELE according to a scale devised for the study that included two grades. Grade 1 was assigned to MELE that had eyelid hairs in contact with the cornea predominantly pointing in one direction (medially or laterally). Grade 2 was assigned to MELE that had eyelid hairs which were in contact with the cornea and were crossing over each other to point in both directions (both medially and laterally) (Fig. 9).

### Sample size calculation

Optimal sample size was determined by performing a power calculation. A confidence level (CI) of 95% was selected, with 5% degree of precision and estimated prevalence of 82.4% (based on the results of a previous study [13]. Power analysis estimated the optimal sample size for this study to be 223 Pugs.

### Statistical analysis

Percentage, mean (+/− *sd*) and median (range) were used to summarise categorical and continuous variables. Age, STT1, MELE (% length), MEUE (% length), CFI, nasal fold width, palpebral fissure width and limbal pigmentation were analysed as continuous variables, whereas sex, neuter status, UK KC registration, coat colour, show dog status, ONW, IIPPMs and distichia were analysed as nominal variables and MELE (grade) as an ordinal variable. Repeated measure logistic regression (to account for correlation between the two eyes from the same pug) was used to assess association of predictors (the abovementioned signalment, breed club registration, ophthalmic, adnexal and facial findings) with detection and severity of PK. Predictors with *P* ≤ 0.10 in the univariable analysis were included in the multivariable analysis, and backward elimination was used for model selection. Two-way interaction between predictors were assessed in the final multivariable model. Predictors with *P* < 0.05 were considered significant. Odds ratio (OR) and its 95% confidence intervals (CI) were reported. Statistical analyses were performed using IBM SPSS software (IBM SPSS Statistics for Macintosh, Version 24.0).

## Supplementary information

**Additional file 1.** Pigmentary keratitis in Pugs in the UK – Examination Form. Examination Record Form.

## Data Availability

The datasets used and/or analysed during the current study are available from the corresponding author on reasonable request.

## Abbreviations

CFI: Craniofacial index
IIPPMs: Iris-to-iris persistent pupillary membranes
KC: Kennel Club
KCS: Keratoconjunctivitis sicca
MELE: Medial entropion of the lower eyelid
MEUE: Medial entropion of the upper eyelid
ONW: Over-nose-wrinkle
OR: Odds ratio
PK: Pigmentary keratitis
*sd*: Standard deviation
STT1: Schirmer tear test 1
UK: United Kingdom
USA: United States of America
