# Supplementary material for: Pigmentary keratitis in pugs in the United Kingdom: prevalence and associated features
Source: BMC Vet Res. 2019 Oct 30;15:384. doi: 10.1186/s12917-019-2127-y (PMC6822449; doi:10.1186/s12917-019-2127-y)
Supplement: Supplementary file 1 — Additional file 1. Pigmentary keratitis in Pugs in the UK – Examination Form. Examination Record Form. [file 12917_2019_2127_MOESM1_ESM.doc]

Pigmentary keratitis in Pugs in the UK – Examination Form

| **HISTORY/EXAMINATION** | **RIGHT EYE** | **LEFT EYE** |
| --- | --- | --- |
| Ophthalmic history (e.g. KCS) |  |  |
| Schirmer Tear Test 1 | ..........mm | ...........mm |
| Medial entropion   - Estimate % of lid length affected - Assess grade | 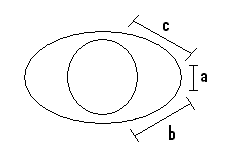  a = .......... % ...........grade  b = .......... % ...........grade  c = .......... % ...........grade | 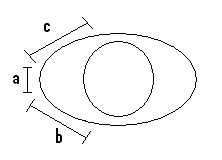  a = .......... % ...........grade  b = .......... % ...........grade  c = .......... % ...........grade |
| Limbal pigmentation   - Estimate number of ‘clock hours’ of the limbus that are pigmented | 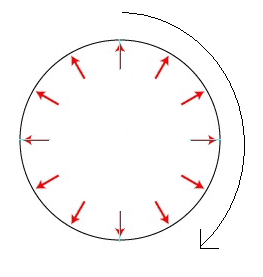  ........ clock hours | 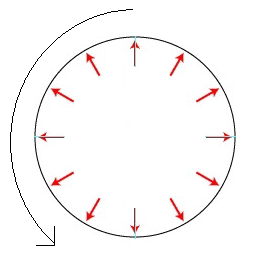  ........ clock hours |
| Corneal pigmentation   - Estimate number of ‘clock hours’ of the cornea that are pigmented - Draw freehand - Other corneal lesions | **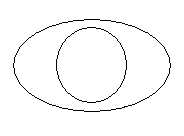**  ........ clock hours | 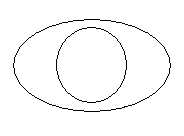  ........ clock hours |
| Iris-iris PPMs (Y/N) |  |  |
| Distichia (Y/N) |  |  |
| Other clinical findings |  |  |

| **ADNEXAL & FACIAL FEATURES** | **RIGHT EYE** | | **LEFT EYE** |
| --- | --- | --- | --- |
| Cranial length (soft measuring tape) | .......... cm | | |
| Muzzle length (soft measuring tape) | .......... cm | | |
| Head width (Vernier callipers at the widest point of the head) | .......... cm | | |
| Presence of over the nose wrinkle (Y/N) |  | | |
| Nasal fold width (Jameson caliper) | .......... mm | .......... mm | |
| Palpebral length (Jameson caliper) | …....... mm | …....... mm | |
